# Imprinted Genes That Regulate Early Mammalian Growth Are Coexpressed in Somatic Stem Cells

**DOI:** 10.1371/journal.pone.0026410

**Published:** 2011-10-19

**Authors:** Jonathan S. Berg, Kuanyin K. Lin, Corinne Sonnet, Nathan C. Boles, David C. Weksberg, Hoang Nguyen, Lowenna J. Holt, Danny Rickwood, Roger J. Daly, Margaret A. Goodell

**Affiliations:** 1 Department of Molecular and Human Genetics, Baylor College of Medicine, Houston, Texas, United States of America; 2 Stem Cells and Regenerative Medicine Center, Baylor College of Medicine, Houston, Texas, United States of America; 3 Interdepartmental Program of Cell and Molecular Biology, Baylor College of Medicine, Houston, Texas, United States of America; 4 Department of Pediatrics, Baylor College of Medicine, Houston, Texas, United States of America; 5 Diabetes and Obesity Research Program, Garvan Institute of Medical Research, Sydney, Australia; 6 Cancer Research Program, Garvan Institute of Medical Research, Sydney, Australia; Stanford University, United States of America

## Abstract

Lifelong, many somatic tissues are replenished by specialized adult stem cells. These stem cells are generally rare, infrequently dividing, occupy a unique niche, and can rapidly respond to injury to maintain a steady tissue size. Despite these commonalities, few shared regulatory mechanisms have been identified. Here, we scrutinized data comparing genes expressed in murine long-term hematopoietic stem cells with their differentiated counterparts and observed that a disproportionate number were members of the developmentally-important, monoallelically expressed imprinted genes. Studying a subset, which are members of a purported imprinted gene network (IGN), we found their expression in HSCs rapidly altered upon hematopoietic perturbations. These imprinted genes were also predominantly expressed in stem/progenitor cells of the adult epidermis and skeletal muscle in mice, relative to their differentiated counterparts. The parallel down-regulation of these genes postnatally in response to proliferation and differentiation suggests that the IGN could play a mechanistic role in both cell growth and tissue homeostasis.

## Introduction

Somatic stem cells are collectively defined by their ability to self-renew and to differentiate to replenish tissue throughout adult-hood. Some somatic stem cells, such as hematopoietic stem cells (HSC), can differentiate into a plethora of cell types, whereas others are much more limited, maintaining a quite restricted cell population in a particular tissue, such as the satellite cells of the muscle. In contrast to embryonic stem cells, somatic stem cells are inherently restricted in their differentiation potential, generally only replenishing the tissue type from which they are derived. Another key feature of many somatic stem cells is that they are generally considered to be quiescent, dividing infrequently, but driven into cycle during periods of tissue regeneration or self-renewal. While this is broadly the case for some canonical stem cells, such as hematopoietic stem cells [1], satellite cells [2], and epidermal stem cells [3], some, such as intestinal stem cells [4] and neural progenitor cells (NPCs) [5] do not fit this stereotype.

Systematic approaches to identify “stemness factors” common to embryonic, neural, and hematopoietic stem cells [6], [7] were unsuccessful [8], possibly owing to the very distinct lifestyle that ES cells possess in comparison to somatic stem cells. Nevertheless, as more somatic stem cell populations have been uncovered over the past decade, the question of whether somatic stem cells, in general, share common regulatory mechanisms has repeatedly been revived. From empirical studies, developmental pathways such as the Wnt and Notch signaling pathway have been shown to impact cell fate decisions in several stem cell types [9], [10], however specific common regulatory genes have still not been uncovered.

Another approach is to determine the factors that make stem cells distinct from their differentiated progeny by comparing the expression profiles of somatic stem cells to those of their differentiated counterparts, for example, of HSCs to their differentiated blood progeny [11]. In this study, we found in HSCs an intriguing enrichment for genes that were regulated by genomic imprinting.

Imprinting is an epigenetic phenomenon in which certain genes are expressed in a monoallelic fashion, depending on their parental origin. Imprinted genes are widely considered to have critical roles in embryonic development [12], [13], and alterations of their expression are responsible for several human genetic syndromes [14]. The mono-allelic expression makes the imprinted genes vulnerable to inactivation through mutation or epigenetic silencing, presumably accounting for their low prevalence, estimated at less than 1% of all human and mouse genes [15]. Nevertheless, genomic imprinting has been maintained throughout mammalian evolution, perhaps as a mechanism for balancing maternal and paternal interests with regard to the growth of offspring, a hypothesis referred to as the “kinship theory” or “conflict hypothesis” [13].

Here, we show a subset of imprinted genes, represented in a so-called imprinted gene network (IGN) [16] that are down-regulated postnatally [17], are predominantly expressed in somatic stem cells, relative to their differentiated progeny. This observation leads to the suggestion, explored here, that the IGN could play a broad role in regulating multiple somatic stem cells.

## Results

### Members of the imprinted gene network (IGN) that are developmentally silenced in somatic tissues remain expressed in long-term hematopoietic stem cells

**Figure 1:**
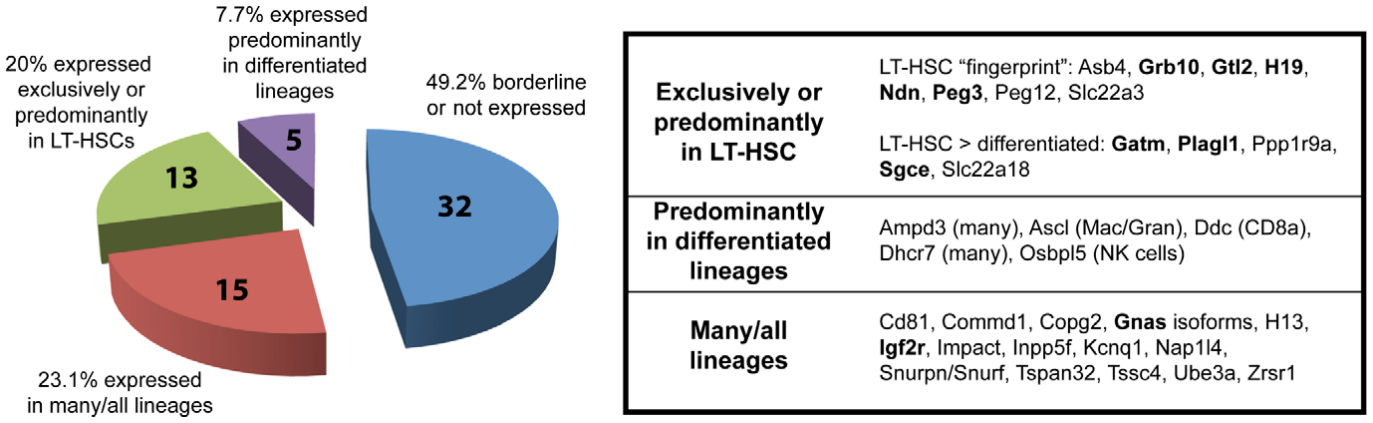
Summary of microarray analysis of imprinted gene expression in hematopoiesis. Microarray data from mouse LT-HSCs and differentiated lineages [11] were analyzed for the expression of known imprinted genes. The chart (left) shows the distribution of genes according to the specificity of their lineage distribution; the individual genes in each expression category are listed to the right (genes previously identified as being part of the IGN, as defined by Varrault et al. [16] are noted in bold-face type). Supplementary information is provided in Table S1, showing the description of the core IGN genes in this study.

Recognition that several imprinted genes were represented in the gene expression profile we had generated for mouse long-term repopulating hematopoietic stem cells (LT-HSCs) [11] led us to ask whether such genes might play a critical role in the regulation of LT-HSCs and perhaps of other adult stem cells as well. Close examination of the data for each of the hematopoietic cell types previously analyzed in our lab by microarray revealed that imprinted genes, although constituting only 65 (∼0.33%) of the ∼20,000 genes represented on our expression microarrays, accounted for 8 (3.2%) of the 253 genes in a stringently annotated list of “fingerprint” genes expressed exclusively in LT-HSCs, a nearly 10-fold enrichment relative to that expected by chance (P<0.0001 by Fisher's exact test). Five other imprinted genes were expressed predominantly in LT-HSCs with much lower expression in a small subset of differentiated lineages. Thus, an unusually high proportion of known imprinted genes are preferentially expressed in LT-HSCs (Figure 1).

**Figure 2:**
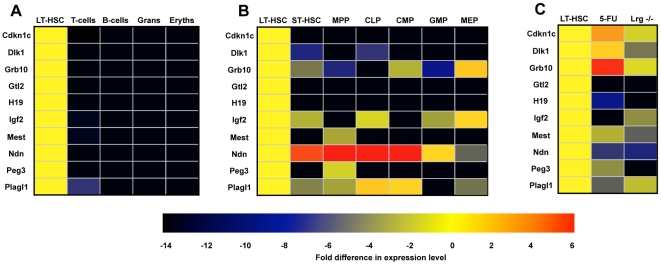
Real-time PCR analysis of imprinted gene expression in hematopoietic cells. Hematopoietic cell populations were isolated, and the expression of 10 core imprinted genes was determined by Q-RT-PCR. The data representing at least two independently isolated biological replicates for each population are shown as heat maps showing the fold difference in gene expression for each cell type compared to LT-HSCs. (A) Terminally differentiated cell populations (T-cells, B-cells, granulocytes, and erythrocytes) vs. HSCs. (B) Hematopoietic progenitor populations vs. LT-HSCs. (C) LT-HSC expression under conditions of acute (5-FU) or chronic (*Lrg47*−/−) proliferative stress vs. quiescence. Proliferating LT-HSCs were collected on day 6 post 5-FU treatment or from Lrg47^−/−^ mice, and imprinted gene expression was determined by Q-RT-PCR.

The imprinted genes exclusively or predominantly expressed in LT-HSCs bear a striking resemblance to a specific subset of imprinted genes referred to as the imprinted gene network (IGN) [16]. First identified through a meta-analysis of mouse gene-expression datasets, this group of genes is thought to be critically involved in the control of fetal and early postnatal growth, becoming down-regulated with the increasing age of the organism and slowing of the somatic growth rate [17]. Although others have reported subsets of this network (Table 1) [18], [19], [20], their link to a formal genetic program was often not recognized. Moreover, a number of the differentially expressed genes identified by our microarray analysis [11] are not part of the IGN as originally defined by Varrault et al [16]. Finally, we examined our previous microarray data for the 58 genes reported by Finkielstain et al. [21] to be down-regulated >3-fold between week-1 and week-4 of postnatal growth in heart, kidney and lung; 26 (44.8%) had flat expression profiles across hematopoietic populations, 13 genes (22.4%) were expressed in many or all lineages, and 10 genes (17.2%) were predominantly expressed in myeloid lineages. Quite strikingly, while the list of 58 genes reported by Finkielstain et al. contains 5 members of the IGN, it contains only 4 non-imprinted genes that are expressed predominantly in LT-HSCs (*Sox4*, *Zfp184*, *Emelin1*, and *Smarca1*). Thus, the genes that are down-regulated with age are not simply enriched for LT-HSC “fingerprint” genes, but are rather enriched for members of the IGN. These findings suggest that a specific group of imprinted genes, critical for embryonic growth but silenced in somatic tissues during early postnatal growth, remain expressed in LT-HSCs and may participate in the regulation of LT-HSC function. To explore this possibility, we selected 10 imprinted genes known to be coregulated during embryonic growth [16], [18], [19], [20], including three with indeterminate microarray expression (*Cdkn1c*, *Igf2*, and *Mest*) that were previously linked to hematopoiesis [22], [23], [24], [25]. Quantitative real-time PCR (Q-RT-PCR) was performed for these genes (*Cdkn1c*, *Dlk1*, *Grb10*, *Gtl2*, *H19*, *Igf2*, *Mest*, *Ndn*, *Peg3*, and *Plagl1*) in LT-HSCs and representative differentiated lineages, which had been purified independently of the original microarray study. This analysis showed that the coexpressed genes were at least 30-fold more abundant in LT-HSCs than in their differentiated progeny, with the exception of *Igf2*, *Mest*, and *Plagl1*, which retained some expression in T-cells (Figure 2A).

**Table 1:** Putative members of the IGN identified in six different studies.

Previous studies						This study				
Gene	Varrault (2006) [16] Microarray meta-analysis	Yan (2003) [18] Muscle regeneration	Dekel (2006) [19] Kidney progenitors	Lui (2008) [17] Downregulated with age	Gabory (2009) [20] Transgenic H19 overexpression	Hematopoietic system (microarray and Q-RT-PCR data)				
						LT-HSCs	Progenitors	Differentiated	Muscle satellite cells	Skin stem cells
Asb4						√		−		
**Cdkn1c**	√	√		√	√	√	−	−	√	√
Dcn	√				√					
**Dlk1**	√		√	√	√	√	√	−	√	√
Gatm	√					√		+/−		
Gnas	√				√	√		√		
**Grb10**	√			√		√	√	−	√	√
**Gtl2**	√			√		√	−	−	√	−
**H19**	√	√		√	√	√	−	−	√	√
**Igf2**	√	√	√	√	√	√	√	−	√	√
Igf2r	√				√	√		√		
Impact						√		+/−		
**Mest**	**√**	**√**	**√**	**√**		**√**	**+/−**	**−**	**√**	**√**
**Ndn**	**√**			**√**		**√**	**√**	**−**	**√**	**√**
Nnat			√							
**Peg3**	**√**	**√**	**√**	**√**	**√**	**√**	**+/−**	−	**√**	**√**
Peg10			√							
Peg12						√		−		
**Plagl1**	**√**	**√**		**√**		**√**	**√**	**+/−**	**√**	**√**
Ppp1r9a						√		−		
Rtl1					√					
Sgce	√					√		+/−		
Slc22a3,						√		−		
Slc22a18						√		−		
Slc38a4	√			√	√					

The ten members of the IGN studied in this manuscript are indicated in bold. Check marks (√) indicate positive identification of IGN members by gene expression assays. Absence of expression is not explicitly indicated except in examples from our study, where differential expression was observed by microarray, or Q-RT-PCR failed to detect amplification (−). Variable expression (observed in some but not all cell types) is indicated (+/−).

We next examined the expression of the genes in minimally differentiated progenitor populations not previously studied with our microarrays, including short-term hematopoietic stem cells (ST-HSCs), multipotent progenitors (MPPs), common lymphoid progenitors (CLPs), common myeloid progenitors (CMPs), myeloid-erythroid progenitors (MEPs), and granulocyte-macrophage progenitors (GMPs). Interestingly, the pattern of gene expression by Q-RT-PCR was more varied among ST-HSCs and progenitors than in LT-HSCs (Figure 2B), suggesting that these imprinted genes may have different roles during the earliest phases of hematopoietic development. For example, expression of *Ndn* is several fold higher in the early progenitor cell populations than in LT-HSCs, which is consistent with a recent report indicating a possible role for Ndn in cell cycle control in hematopoietic stem and progenitor cells [26].

Monoallelic expression dependent on the parent of origin is a hallmark of imprinted genes. However, certain imprinted genes become biallelically expressed in adult tissues, and we are unaware of any studies that have analyzed imprinting in somatic stem cells. We therefore determined the mode of expression of *Dlk1*, *Gtl2*, *H19*, *Igf2* and *Peg3* in LT-HSCs isolated from the F1 progeny of Castaneous and C57Bl/6 parents (Figure S1A). Analysis of coding SNPs allowed us to identify the parent-of-origin for the transcripts of these five genes, showing that the expressed allele was concordant with the reported imprinting pattern for each gene, confirming that monoallelic expression is generally retained in LT-HSCs (Figure S1 and Table S2).

### The abundance of IGN gene expression correlates with functional stem cell properties

Contrasting roles have been attributed to paternally and maternally biased alleles in diverse cellular processes [27]. Experimental disruption of imprinting can induce dramatic phenotypic changes in growth leading to malignant transformation [28], while imprinted genes are frequently dysregulated in tumorigenesis [19], [29], including myeloproliferative diseases [23], [30], [31] (summarized in Table S1). Since one of the hallmarks of stem cells is their capacity to replenish a tissue by responding to short-term and long-term signals, we investigated whether expression of our core group of imprinted genes might be involved in acute or chronic changes (or both) in the LT-HSC response to proliferative stress, by assessing their expression under two distinct conditions that mimic an acute response to injury and chronic overstimulation, respectively (Figure 2C).

We first used 5-fluorouracil (5-FU) to ablate cycling short-term bone marrow progenitor cells, and thus to stimulate transient proliferation of LT-HSCs (an injury from which the cells completely recover) [32]. At 6 days after 5-FU treatment, when the cells are maximally proliferative [32], *Gtl2* was undetectable, *Igf2* and *Peg3* were downregulated more than 3-fold, and *Cdkn1c* expression was decreased 2-fold, while *Grb10* showed a 2.8-fold increase in expression. We next investigated the effect of chronic perturbation of LT-HSCs using mice deficient for *Irgm* (encoding Lrg47, an interferon-inducible GTPase), which have decreased numbers of LT-HSCs that exhibit sustained high proliferation, resulting in failure to engraft in bone marrow transplantation assays [33]. By Q-RT-PCR analysis, *Gtl2*, *H19*, and *Peg3* were undetectable, *Dlk1*, *Mest* and *Ndn* were downregulated >4-fold, and *Igf2* and *Plagl1* showed modest decreases in expression. *Gtl2* is a host transcript for a number of miRNAs, and has recently been shown to inhibit induced pluripotential stem cell formation when aberrantly silenced [34]. We verified that multiple miRNAs from the *Gtl2* locus are also expressed in LT-HSCs and several of them are strikingly down-regulated in LT-HSCs deficient for *Irgm* (Figure S2).

Thus, two conditions that disrupt LT-HSC homeostasis (one pharmacologic and one genetic) perturb the expression of a central group of imprinted genes known to be involved in embryonic and early postnatal growth. Acute proliferative stress perturbs the expression of only a subset of the imprinted genes, while chronic proliferation due to *Irgm* deficiency (in which LT-HSC function is more severely compromised) correlates with greater alteration of the imprinted genes, including several miRNAs. Whether the above findings indicate a direct mechanistic relationship between the change in proliferative status and altered gene expression or merely capture genetic epiphenomena associated with an altered LT-HSC state is unclear.

We hypothesize that the retained expression of the imprinted genes in hematopoietic stem/progenitor cells allows a poised state of growth control that permits rapid response to growth stimulatory signals in this special cell population. This appears to be a different mechanism than the control of proliferation in differentiated hematopoietic lineages, since our previous microarrays included proliferating (e.g. activated T-cells and nucleated erythroid lineages) and non-proliferating cells that would have retained some potential for division (e.g. quiescent T-cells, and monocytes). If the IGN were only correlated with quiescence versus proliferation, we would expect to see a different pattern than what was observed.

### Imprinted gene expression is highly enriched in multiple somatic stem cells, including murine muscle and epidermal, and human epidermal and hematopoietic stem cells

**Figure 3:**
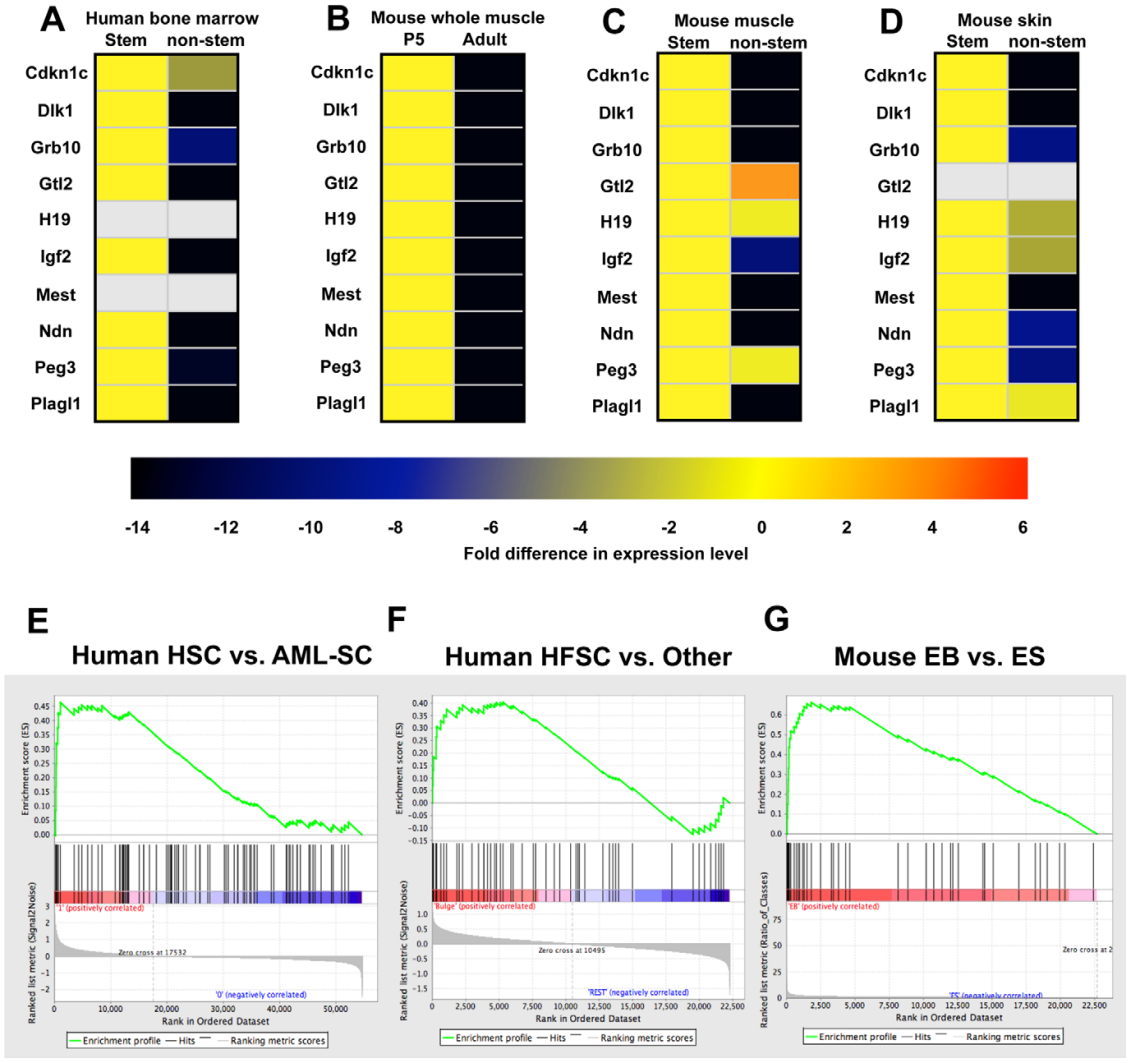
IGN expression is retained in mouse and human somatic stem cell populations but not embryonic stem cells. The expression of imprinted genes was compared in (A) human bone marrow CD34+ vs. CD34− cells, (B) mouse whole muscle cells during skeletal muscle maturation from P5 to adulthood, (C) quiescent muscle satellite cells (SCs) vs. whole muscle (non-SCs), and (D) mouse skin stem cells vs. keratinocytes (non stem cells). The data representing at least two independently isolated biological replicates for each population are shown in heat maps representing fold differences in expression compared to the reference sample (yellow). Grey shading represents RT-PCR probes that failed to amplify in either population. (E–G) GSEA of gene expression in three of the cell population comparisons is shown in Table 1. Each example shows enrichment for the imprinted gene set, with an FDR q-value ≤5% and an enrichment P-value≤0.03. EB, embryoid bodies; ESC, embryonic stem cells; HFSCs, hair follicle stem cells. Supplementary information is provided in Figure S1, showing the monoallelic expression nature of genes such as Dlk1, Gtl2, H19, Igf2, and Peg3 in mouse HSCs, as well as Table S2, showing the SNPs used for the analysis of monoalleic expression.

The imprinted genes analyzed here represent 10 of the 11 imprinted genes identified in a study that used microarrays to detect genes that were downregulated in murine whole kidney, heart, and lung from birth to maturity [17]. This finding, combined with our data from LT-HSCs, suggests that our group of imprinted genes are broadly expressed in embryonic and rapidly growing postnatal tissues, but then becomes restricted to tissue-specific stem cells, which retain the license to proliferate and differentiate to meet specific organismal demands. To test this concept, we studied additional tissues and stem cells. After confirming selective expression in human CD34+ stem cells from whole bone marrow (Figure 3A) and cord blood (data not shown) we found that all of the 10 genes tested were highly expressed in whole mouse postnatal day-5 (P5) skeletal muscle and were downregulated >100-fold in whole adult (6- to 8-week-old) muscle (Figure 3B). However, each imprinted gene was expressed in muscle satellite cells isolated from either P5 or adult mice with relatively few differences (data not shown). Importantly, except for *Peg3*, *H19*, and *Gt12*, the imprinted genes were expressed at least 10-fold more highly in quiescent satellite cells than in whole muscle (Figure 3C). In skin, 9 of the 10 imprinted genes were detected in epidermal stem cells (CD34^+^ integrin α6^+^ keratinocytes), with *Cdkn1c*, *Dlk1*, *Grb10*, *Mest*, *Ndn* and *Peg3* showing at least 8-fold higher expression levels in stem vs. non-stem cells (Figure 3D). These results clearly demonstrate that the pattern of imprinted gene expression is largely retained in human hematopoietic stem cells, as well as two other murine stem cell compartments. The minor variability of this pattern among different somatic stem cells could be due to cell type-dependent differences in the composition of the proposed regulatory network, or to heterogeneity within the muscle satellite cell and skin stem cell samples (which may represent a mixture of long-term and short-term stem and progenitor cells).

To understand whether the preferential expression of IGN genes in stem cells are preserved in other somatic stem cells, we used Gene Set Enrichment Analysis (GSEA) to assess the expression of imprinted genes among other datasets available via the NCBI Gene Expression Omnibus (Table 2). This method detected enrichment for 24 imprinted genes (16 previously assigned to an IGN [16] as well as 8 identified by our microarray analysis [11]) in LT-HSCs compared to differentiated hematopoietic cells, both in our previous microarrays and an independently derived set of genes expressed during mouse hematopoiesis [35]. We also analyzed human microarray datasets and found that this set of imprinted genes was strikingly enriched in normal human HSCs compared to a putative leukemia stem cell population (AML0-SCs; Figure 3E) [36] and in human hair follicle stem cells (Figure 3F) [37]. Intriguingly, GSEA also revealed enrichment of imprinted gene expression in mouse embryonic fibroblasts (MEFs) compared to embryonic stem (ES) cells and neural progenitor cells (NPCs) [5]. Although NPCs are considered somatic stem cells, they are most important during embryonic development and do not share more than a few of the hallmarks of other somatic stem cells (such as being generally quiescent and participating in lifelong renewal of tissues). The reason for the lack of enrichment of imprinted genes in NPCs is unclear, but may simply reflect a different mechanism of regulation in this distinct type of somatic stem cell. Finally, when ES cells were allowed to differentiate in vitro [38], the expression of imprinted genes was higher in the embryoid bodies (EBs; Figure 3G), indicating that these genes regulate growth potential in the embryo but are not, as a group, part of the pluripotency program executed by ES cells, even though some individual members of the network may be expressed in, and even important for, ES cells. These results reveal an important distinction between ES and adult stem cells and could explain why a network of coexpressed imprinted genes was not identified as an integral component of the stem cell molecular signature in earlier studies [6], [7].

**Table 2:** Enrichment of imprinted genes in somatic stem cell populations from published gene expression studies.

Dataset	GSEA Comparison	IGN gene set enrichment	Enrichment score	NominalP-value^a^	FDR q-value	GEO accession	PMID
**MOUSE**							
HSC vs. 9 differentiated hematopoietic lineages [11]	HSC vs. all lineages	HSC	0.525	0.000	0.000	GSE6506	18371395
HSC vs. MPP and differentiated lineages [35]	HSC vs. (MPP+CD45+)	HSC	0.498	0.034	0.034	none	15989959
ES, MEF, NPC [5]	MEF vs. (ESC+NPC) NPC vs. ESC NPC vs. (ESC+MEF) ES vs. (MEF+NPC)	MEF n.s. n.s. n.s.	0.869	0.000	0.054	GSE8024	17603471
ESC differentiated into EBs [38]	ESC vs. EB	EB	0.663	0.000	0.045	GSE3223	15763554
**HUMAN**							
Bone marrow HSC vs AML stem cells [36]	HSC vs. AML	HSC	0.4663	0.034	0.043	GSE17054	19218430
Skin bulge cells vs non-bulge cells [37]	Bulge vs. nonbulge	Bulge	0.404	0.008	0.0327	GSE3419	16395407

Datasets from the Gene Expression Omnibus (GEO) that included stem cells together with a comparator population were used to examine enrichment for the IGN using Gene Set Enrichment Analysis (GSEA) with a custom imprinted-gene gene set. As originally defined [16], the IGN includes 16 genes, but for this analysis we also included additional imprinted genes that were specific to the LT-HSCs in our study [11]. This combined IGN-HSC list includes *Asb4*, *Cdkn1c*, *Dcn*, *Dlk1*, *Gatm*, *Gnas*, *Grb10*, *Gtl2*, *H19*, *Igf2*, *Igf2r*, *Impact*, *Mest*, *Ndn*, *Nnat*, *Peg3*, *Peg10*, *Peg12*, *Plagl1*, *Ppp1r9a*, *Sgce*, *Slc22a3*, *Slc22a18*, and *Slc38a4*.

(a) The nominal P-value for all tests indicating 0.000 is <0.0001 (1000 permutations were used in each test). HSC, hematopoietic stem cells; MPP, multipotential progenitors; NPC, neural progenitor cells; ESC, embryonic stem cells; EB, embryoid bodies; AML, acute myeloid leukemia; FDR, false discovery rate; GEO, gene expression omnibus; PMID, pubmed ID; ns, not-significant.

## Discussion

**Figure 4:**
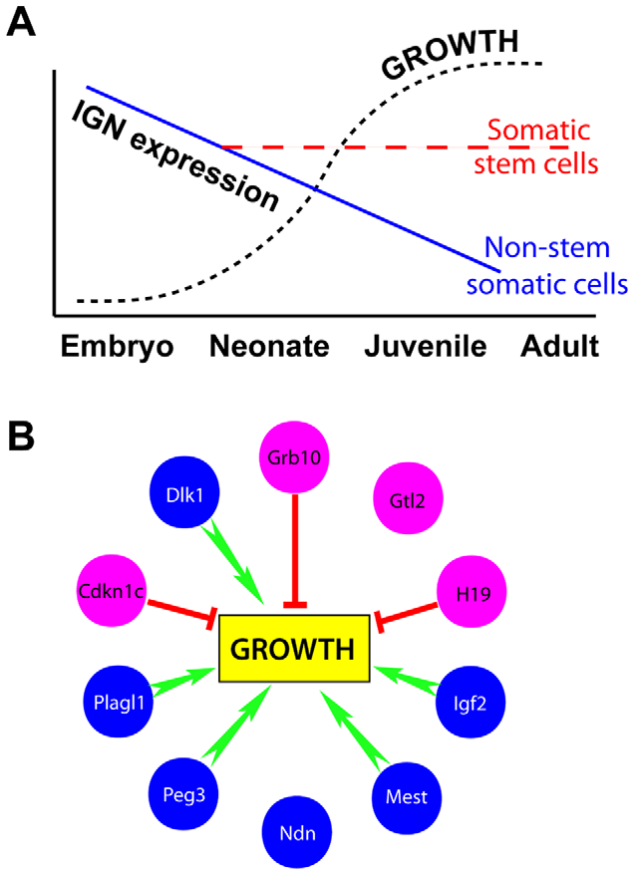
Models of IGN during development. (A) IGN members are highly expressed during embryogenesis but are downregulated in whole tissue as growth proceeds, while their expression is maintained in adult stem cell compartments. (B) Representation effects of the IGN on growth. Maternally expressed genes are depicted in pink and paternally expressed genes in blue. Overall effect on growth as determined by transgenic or knockout animal models is shown in green (promotion) or red (inhibition).

Many tissues undergo lifelong replenishment by rare specialized adult stem cells, which are generally quiescent, occupy unique niches, and respond rapidly to injury in order to maintain the size of a particular organ or tissue. We previously observed that a group of developmentally important imprinted genes are preferentially expressed in mouse long-term hematopoietic stem cells (LT-HSCs) compared with their differentiated counterparts [11]. Here we show that subsets of these “fingerprint” genes are core members of a network of coregulated imprinted genes, which have been implicated in the control of embryonic and early postnatal growth [16], [17]. These genes are expressed in mouse stem and progenitor cells of the hematopoietic system, skin, and skeletal muscle, and their expression is significantly lower in the differentiated progeny of these cells and in perturbed LT-HSCs. Human hematopoietic stem cells also preferentially express these genes. The imprinted genes identified in this study are highly expressed during embryonic and early postnatal life, silenced as the organism nears its adult body size [17], but remain expressed in at least three types of somatic stem cells (Figure 4A). We speculate that these imprinted genes represent a special subset of the LT-HSC “fingerprint” genes and that perhaps other somatic stem cells likewise express a larger set of “stemness” genes comprised of lineage-specific developmental regulatory genes in addition to the smaller subset of IGN members shared with other somatic stem cells.

These data for the first time implicate genes associated with embryonic and early postnatal growth [17] in regulation of somatic stem cells. Such a parallel role may not be entirely surprising, as the requirements for rapid proliferation and expansion of somatic stem cells in response to external cues may be similar to those imposed by embryonic and early postnatal development. Interestingly, maternally-expressed and paternally-expressed genes with opposing roles in growth regulation are represented in the IGN, suggesting a balance in growth-promoting and growth-restraining forces within the network, perhaps at play in somatic stem cells (Figure 4B and Table S1). The proteins encoded by these imprinted genes are sufficiently diverse to influence stem cells via a variety of strategies, including endocrine/paracrine signaling and signal transduction, developmental morphogenesis, regulation of apoptosis, and cell cycle inhibition (Table S1), while *H19* and *Gtl2* noncoding RNA transcripts harbor micro-RNAs [39], [40] that could modulate the expression of large numbers of target genes. Interestingly, the tumor suppressor p53, which was recently implicated in the regulation of HSC quiescence [41], appears to be functionally linked to several members of the IGN [41], [42], [43], [44], [45], again suggesting a central role for imprinted genes in the regulation of cellular growth. Together, these findings link a putative imprinted gene network with the regenerative capacity of adult stem cells, offering a possible mechanism for influencing organismal size by coordinating post-natal growth potential.

A “network” implies an interconnected group of genes whose individual changes in expression can influence the expression or function of the remaining genes. Indeed, overexpression of *Plagl1* (*Zac1*) in the Neuro2a cell line or its knockout in liver resulted in altered expression of some, but not all, IGN members [16]. Similarly, *H19* expression appears to have a role in regulating the expression of certain IGN members [20], [46]. Whether these imprinted genes indeed function as a network *in vivo*, and what their individual roles are in stem cells, will have to be determined. The complementary and contrasting functions of the proteins encoded by the group of imprinted genes examined in this study may pose challenges to determining their function in stem cells, because alterations in the expression of single genes could be buffered by the actions of others. Indeed, one might predict that members of a coregulated network would work together to provide some level of redundancy, such that arbitrary elimination of a single gene would not be expected to disrupt the robustness of the entire program. Thus, manipulation of different combinations of imprinted genes, via double knock-outs or pools of siRNAs, may be needed to show major effects on somatic stem cell function. This concept may be borne out by the somewhat modest impact of KO of some individual members of the IGN, such as necdin [26].

A key question for the future will be the nature of the coordinated regulation of imprinted genes. A number of studies have suggested they may exist in proximity in the nucleus [47], and a recent report indicates that there could be a link to chromatin regulators such as ATRX, MeCP2, and cohesin [48]. In addition, the polycomb repressive complex 1 protein, Bmi-1 may contribute to their regulation [49]. Further studies may reveal whether IGN member expression is truly interdependent, or controlled by some yet unknown master regulator.

An additional question is whether IGN expression in somatic stem and progenitor cells exists simply because of the collective functions of IGN members, or whether there is a unique contribution of imprinting in this case. Many investigators have speculated about the evolutionary conservation of imprinting, and one hypothesis put forward recently (the “Rheostat model”) is that genomic imprinting could confer evolutionary advantage if imprinted genes have evolved mechanisms for generating quantitative hypervariability in expression levels that can mediate phenotypic differences between individuals in a population [50]. According to this model, quantitative hypervariability in the expression of imprinted genes would be highly adaptive since alleles could be masked by imprinting (and thus protected from selection) and yet emerge and propagate in a population under favorable conditions. Genes controlling embryonic and postnatal growth, as well as adult stem cell function would be ideal candidates for this type of “rapid and reversible evolution” of phenotypes within a population, and thus perhaps expression of the IGN is retained in somatic stem cells in part due to the unique characteristics of genomic imprinting.

Finally, it remains to be determined whether our findings are broadly generalizable to other somatic stem cell populations. The three stem cell types studied here, hematopoietic, skin, and muscle, share similarities in that they are generally quiescent and require very specific interactions with a niche environment. A recent study generated a GFP-knock-in to the Peg3 locus and found remarkably stem cell-specific expression of Peg3 in multiple somatic tissues including skeletal muscle, bone marrow, CNS, testis, and others [51]. Another recent paper suggests components of the IGN may be present in lung stem cells [49]. However, it is possible that other somatic stem cell types, perhaps those with high turnover (e.g. intestinal stem cells [52]) may have quite different patterns of imprinted gene expression, or not share elements of this network at all.

It is intriguing that paternally and maternally expressed genes have opposing roles in fetal and placental development, forming the basis of the parental “conflict hypothesis” [12]. Stem cells could be viewed as having a similar conflict between growth promotion and restraint, regulated by intrinsic gene expression programs and external cues from the niche. This leads us to speculate that the diverse functional roles of imprinted genes in coregulated networks might have been co-opted during evolution to support the complexity of stem cell regulation of tissue homeostasis.

## Methods

### Analysis of microarray data

A list of mouse genes with known imprinting status was generated using four well-curated catalogs of imprinted genes (www.otago.ac.nz/IGC [15]; http://www.geneimprint.com/site/genes-by-species.Musmusculus; WAMIDEX: https://atlas.genetics.kcl.ac.uk [53]; http://www.mousebook.org/catalog.php?catalog=imprinting). We excluded genes with evidence against imprinting in mouse, no report of imprinting status in mouse, or conflicting data. Genes classified as imprinted by three of the four catalogs were included in the analysis. Genes with multiple or overlapping transcripts (eg., Gtl2/anti-Rtl/Rian/Mirg; Snurpn/Snurf; Gnas isoforms) were considered as a single “gene” for the purpose of probe annotation. By these criteria, 65 known mouse imprinted genes are represented by probes on the Affymetrix MOE430.2 microarrays used in previous expression profiling experiments. We then stringently hand-annotated the published list of genes specifically expressed in LT-HSCs (http://franklin.imgen.bcm.tmc.edu/loligag/lf.php), by removing duplicate probes and probes of unclear or conflicting significance (largely intronic probes or short, unannotated, unspliced transcripts; or genes represented by multiple probes having disparate microarray results). The final list contained 253 genes with high-quality evidence for specific expression in LT-HSCs.

### Mice

Wild-type C57Bl/6 mice were bred at the AALAC-accredited animal facility (Animal Welfare Assurance Number A3823-1) at Baylor College of Medicine (BCM, Houston, TX). All procedures were approved by the BCM Institutional Animal Care and Use Committee (IACUC) and approved under protocol AN2234. For 5-FU treatment, mice were injected intraperitoneally with a 150 mg/kg dose of drug (Sigma) and killed at day 0 (untreated) or day 6 after injection. *Lrg47* ^−/−^ mice were maintained in the BCM animal facility as per our previous report [30]. All mice were treated according to an approved animal use protocol.

### Isolation of cell populations

Skin stem cells were isolated from adult mice by trypsin floating of the back skins overnight, followed by scraping and cell straining to achieve a single cell suspension, and then exposed to directly conjugated primary antibodies against α6 integrin (CD49f, Pharmingen) and CD34 for 30 minutes on ice, followed by washing in PBS. Epidermal cells were gated for single events and viability, and then sorted according their expression of α6-integrin and CD34. Hematopoietic and skin populations were purified with a MoFlo sorting flow cytometer (Dako) or FACSAria (BD Biosciences). Muscle satellite cells were obtained from freshly isolated tissue, minced in ice-cold Hanks Balanced Salt Solution (HBSS), and enriched by dissociation followed by a Percoll gradient separation [54]. Hematopoietic stem and progenitor cells were obtained from whole bone marrow and isolated by fluorescence-activated cell sorting (FACS) according to protocols used routinely in our lab [11], [55]. LT-HSCs were selected either on the basis of the Hoechst efflux “side population” (SP) with cell surface staining for c-Kit^+^ and Sca-1^+^, and negative staining for linage markers (Lin^−^: CD4, CD8, B220, Mac-1, Gr-1, and Ter-119), collectively termed “KSL”, or positive staining of CD150, negative for CD48 with KLS. For the 5-FU experiment, Mac-1 was left out of the lineage cocktail since this epitope emerges during proliferation. B-lymphocytes (CD19^+^, 33D1^−^) and T-lymphocytes (CD4+ or CD8+, CD25^−^, CD69^−^) were isolated from spleen while granulocytes (Gr-1^+^, 7/4 clone^+^, CD2^−^, CD5^−^, B220^−^, F4/80^−^, ICAM-1^−^, Ter-119^−^) and nucleated erythrocytes (Ter119^+^, CD3^−^, CD4^−^, CD8^−^, Mac1^−^, Gr1^−^, B220^−^) were isolated from WBM. ST-HSCs (non-SP, KSL, CD34^high^, Flk2^low^) and MPPs (non-SP, KSL, CD34^high^, Flk2^high^) were isolated from Hoechst-stained WBM. CLPs (KSL, Il7rα^+^) were obtained from WBM as previously described [56]. The myeloid progenitors were contained within the Kit^+^, Il7rα^−^, Sca-1^−^ and Lin^−^ population from WBM and further subsetted into CMPs (CD34^+^CD16/32^−^), MEPs (CD34^−^CD16/32^−^), and GMPs (CD34^+^CD16/32^+^) [57]. Flow cytometry antibodies were purchased from BD Bioscience and ebiosciences: APC-conjugated CD34, APCcy7-conjugated c-Kit, PEcy7-conjugated CD150, PEcy5 conjugated Lineage antibodies (CD4, CD8, B220, Ter119, Mac-1, and Gr-1) and FITC-conjugated CD48. Cells were analyzed using an LSRII (Becton Dickinson).

### RNA isolation and Q-RT-PCR analysis

Total RNA was isolated from hematopoietic, muscle, and skin cells using RNAqueous (Ambion), followed by DNaseI treatment (Invitrogen) and first-strand synthesis using SuperScript III and priming with random hexamers. Q-RT-PCR was performed with pre-validated Taqman probe sets (Applied Biosystems) on a 7300 Real-Time PCR system for 50 cycles. An internal 18s rRNA control was included in every reaction for normalization. The threshold cycle was determined with software provided by the manufacturer, and expression was measured for each assay relative to the 18s rRNA internal standard (ΔCt). Assays were performed in triplicate (technical replicates) and each experiment was performed in at least two biological replicates. Relative expression between two cell populations was calculated by subtracting the ΔCt values (ΔΔCt). Fold differences were calculated as 2∧(ΔΔCt) when ΔΔCt>0 or −(2∧(−ΔΔCt)) when ΔΔCt<0. A true ΔΔCt cannot be calculated when amplification is not detected for a given sample, so in those cases the maximum cycle number (50) was used to provide a lower boundary of fold change. Heat map data represent the fold change between two populations from at least two independent biological samples.

### Gene Set Enrichment Analysis (GSEA)

The Gene Expression Omnibus (GEO) was searched for datasets that included stem cells together with a comparator population that could be considered differentiated. Imprinted gene lists were then constructed and a chip-to-chip method was used to obtain probes sets for all relevant chips. GSEA was used to examine the enrichment for the imprinted gene network together with the additional imprinted genes specific to HSCs. This analysis was performed with 1000 permutations for each test. We used phenotype permutations for all datasets except Goodell and Vogel [11], [37], for which geneset permutations were required due to some samples having only two replicates.

### Monoallelical expression of the imprinted genes in LT-HSCs

Male Castaneous mice (Cast) were crossed with female C57BL/6 mice (B6) so that the F1 progeny would be heterozygous at SNPs that differ between the two strains (Figure S1A). The Perlegen browser (http://mouse.perlegen.com/mouse/browser.html) was used to identify coding region single nucleotide polymorphisms (SNPs) between the Castaneous and C57BL/6 strains [58] and found SNPs in the coding regions of 3 imprinted genes (*Dlk1*, *Gtl2*, and *Peg3*). Castaneous SNPs were also reported in *H19* and *Igf2* and we adapted the SNuPE assays for these genes [59] using sequence-based detection. SNPs and primers are summarized in Table S2. Genomic DNA from both parental strains and F1 progeny were used to confirm detection of the different alleles (data not shown).

## Supporting Information

Figure S1 **Imprinted genes are monoallelically expressed in LT-HSCs.** Certain imprinted genes become biallelically expressed in adult tissues, prompting us to determine the mode of expression of *Dlk1*, *Gtl2*, *H19*, *Igf2* and *Peg3* in LT-HSCs isolated from the F1 progeny of Castaneous and C57Bl/6 parents. Analysis of coding SNPs allowed us to identify the parent-of-origin for the transcripts of these five genes, showing that the expressed allele was concordant with the reported imprinting pattern for each gene, confirming that monoallelic expression is generally retained in LT-HSCs. (A) Total RNA was isolated from LT-HSCs obtained from F1 progeny of Castaneous and C57BL/6 parents. cDNA fragments spanning these sites were amplified by PCR and sequenced. (B–F) Sequence traces for 5 members of the IGN were analyzed and found to be consistent with monoallelic expression of the transcripts, in agreement with the reported imprinting status of these genes.
(TIF)

Figure S2 **Downregulation of Gtl2 in LT-HSCs correlates with decreased expression of miRNAs within the Gtl2 locus.** *Gtl2* is an intriguing maternally-expressed noncoding RNA that is thought to result in a very long transcript encompassing two microRNA clusters (*anti-Rtl1* and *Mirg*) and a C/D snoRNA cluster (*Rian*) [40], [60], [61]. Intriguingly, Gtl2 displays a striking transcriptional profile in LT-HSCs following treatment with 5-FU (A) and was one of the most highly down-regulated genes in a microarray transcriptional profiling experiment comparing wild type and *Lrg47* ^−/−^ LT-HSCs [62]. Since *Gtl2* is thought to function as a host transcript for multiple miRNAs, we again used Q-RT-PCR to analyze expression of several mature miRNAs predicted to be processed from this long transcript (B). Given that Gtl2 is strongly downregulated in *Lrg47* ^−/−^ LT-HSCs, we compared expression of miRNAs in LT-HSCs from Lrg47−/− mice and their wild-type littermate controls. Indeed, mmu-miR-127 and mmu-miR-337 (encoded within the anti-*Rtl1* transcript), both displayed decreased expression conservatively estimated at >1000-fold, while mmu-miR-134 and mmu-miR-494 (encoded within the *Mirg* cluster) showed >1000-fold and >100-fold decreased expression, respectively. These results support the idea that the *Gtl2* transcript serves as a substrate for miRNA processing in LT-HSCs and indicate that expression of several mature miRNAs in this region are exquisitely downregulated in the abnormally proliferative LT-HSCs from *Lrg47* ^−/−^ mice. Three other miRNAs within this region (mmu-miR-673 and mmu-miR-370 flanking the core *anti-Rtl1* cluster, and mmu-miR-409 at the distal end of the *Mirg* cluster) did not exhibit statistically significant differences in expression, suggesting that there may be tissue-specific cleavage of mature miRNAs from this region. (A) Microarray profiling of gene expression in LT-HSCs following 5-FU treatment revealed that Gtl2 demonstrates a characteristic pattern of down-regulation (maximal at day 4–6 post 5-FU) and recovery (by day 10). (B) The Gtl2 non-coding transcript harbors two clusters of micro-RNAs (anti-Rtl1 and Mirg) and a cluster of sno-RNAs (Rian). Real-time PCR analysis of miRNA expression in wild type and *Lrg47*−/− LT-HSCs revealed strikingly decreased levels of four out of seven miRNAs examined. Fold change in *Lrg47*−/− relative to wild-type is indicated for miRNAs with significant (p<0.05) changes in expression. n.s. = non-significant. This schematic diagram of the Gtl2 locus is modeled after Lin et al. (2003) [63].
(TIF)

Table S1 **Summary descriptions of 10 core members of the IGN.**
(PDF)

Table S2 **SNPs used for analysis of monoallelic expression in F1 progeny of Castaneous male and C57BL/6 female mice.**
(PDF)
