# Supplementary figures and images for: Imprinted Genes That Regulate Early Mammalian Growth Are Coexpressed in Somatic Stem Cells
Source: PLoS One. 2011 Oct 19;6(10):e26410. doi: 10.1371/journal.pone.0026410 (PMC3198398; doi:10.1371/journal.pone.0026410)

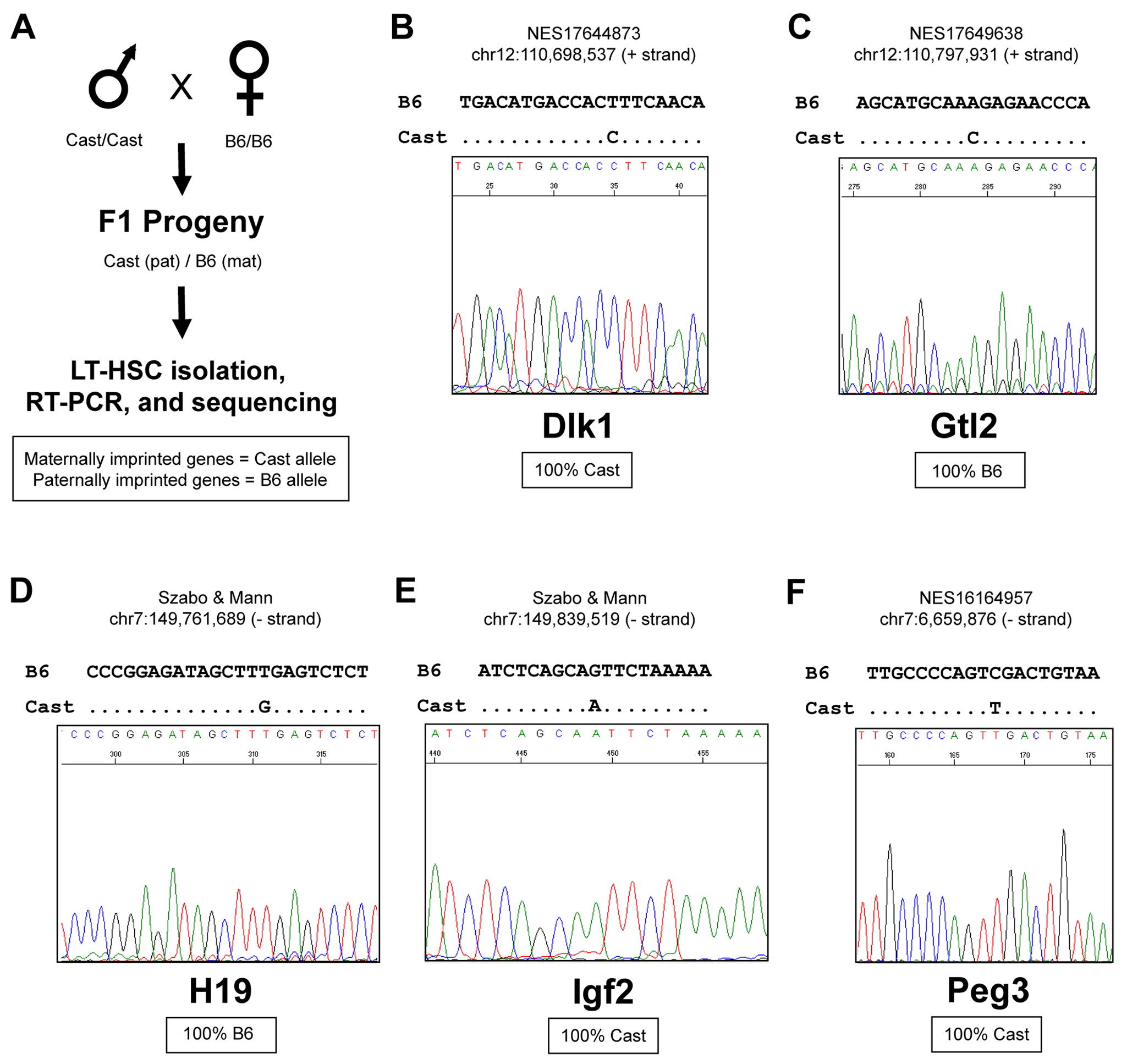

Supplement: Figure S1 — Imprinted genes are monoallelically expressed in LT-HSCs. Certain imprinted genes become biallelically expressed in adult tissues, prompting us to determine the mode of expression of Dlk1, Gtl2, H19, Igf2 and Peg3 in LT-HSCs isolated from the F1 progeny of Castaneous and C57Bl/6 parents. Analysis of coding SNPs allowed us to identify the parent-of-origin for the transcripts of these five genes, showing that the expressed allele was concordant with the reported imprinting pattern for each gene, confirming that monoallelic expression is generally retained in LT-HSCs. (A) Total RNA was isolated from LT-HSCs obtained from F1 progeny of Castaneous and C57BL/6 parents. cDNA fragments spanning these sites were amplified by PCR and sequenced. (B–F) Sequence traces for 5 members of the IGN were analyzed and found to be consistent with monoallelic expression of the transcripts, in agreement with the reported imprinting status of these genes. (TIF) [file pone.0026410.s001.tif]

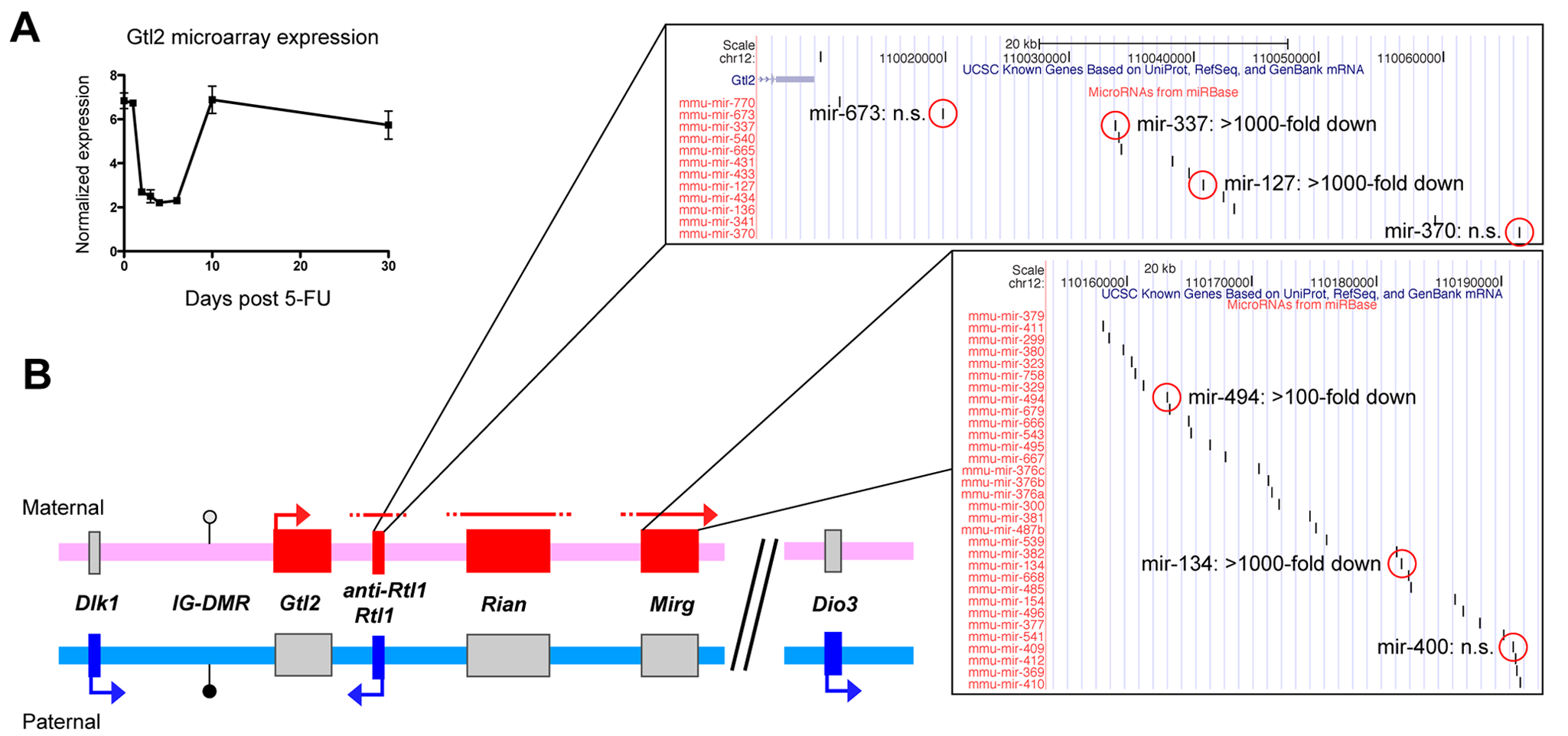

Supplement: Figure S2 — Downregulation of Gtl2 in LT-HSCs correlates with decreased expression of miRNAs within the Gtl2 locus. Gtl2 is an intriguing maternally-expressed noncoding RNA that is thought to result in a very long transcript encompassing two microRNA clusters (anti-Rtl1 and Mirg) and a C/D snoRNA cluster (Rian) [40], [60], [61]. Intriguingly, Gtl2 displays a striking transcriptional profile in LT-HSCs following treatment with 5-FU (A) and was one of the most highly down-regulated genes in a microarray transcriptional profiling experiment comparing wild type and Lrg47 −/− LT-HSCs [62]. Since Gtl2 is thought to function as a host transcript for multiple miRNAs, we again used Q-RT-PCR to analyze expression of several mature miRNAs predicted to be processed from this long transcript (B). Given that Gtl2 is strongly downregulated in Lrg47 −/− LT-HSCs, we compared expression of miRNAs in LT-HSCs from Lrg47−/− mice and their wild-type littermate controls. Indeed, mmu-miR-127 and mmu-miR-337 (encoded within the anti-Rtl1 transcript), both displayed decreased expression conservatively estimated at >1000-fold, while mmu-miR-134 and mmu-miR-494 (encoded within the Mirg cluster) showed >1000-fold and >100-fold decreased expression, respectively. These results support the idea that the Gtl2 transcript serves as a substrate for miRNA processing in LT-HSCs and indicate that expression of several mature miRNAs in this region are exquisitely downregulated in the abnormally proliferative LT-HSCs from Lrg47 −/− mice. Three other miRNAs within this region (mmu-miR-673 and mmu-miR-370 flanking the core anti-Rtl1 cluster, and mmu-miR-409 at the distal end of the Mirg cluster) did not exhibit statistically significant differences in expression, suggesting that there may be tissue-specific cleavage of mature miRNAs from this region. (A) Microarray profiling of gene expression in LT-HSCs following 5-FU treatment revealed that Gtl2 demonstrates a characteristic pattern of down-regulation (maximal at day [file pone.0026410.s002.tif]
