# Supplementary material for: Imprinted Genes That Regulate Early Mammalian Growth Are Coexpressed in Somatic Stem Cells
Source: PLoS One. 2011 Oct 19;6(10):e26410. doi: 10.1371/journal.pone.0026410 (PMC3198398; doi:10.1371/journal.pone.0026410)
Supplement: Table S1 — Summary descriptions of 10 core members of the IGN. (PDF) [file pone.0026410.s003.pdf]

Table S1: Summary descriptions of ten core IGN members analyzed in this study

Gene	Expressed allele	Gene function	Mouse phenotype	Putative role in hematopoiesis or hematopoietic malignancy	Refs
**Cdkn1c**	Maternal	Encodes p57(Kip2), a cyclin-dependent kinase inhibitor. In vitro evidence suggests that p57 inhibits the activity of specific cyclin/CDK complexes. Additional functions include regulation of the cytoskeleton and promotion of apoptosis.	KO: Multiple malformations, neonatal lethal	Highly expressed in quiescent LT-HSCs but downregulated in proliferative short-term HSCs. In human CD34+ cord blood cells, *CDKN1C* is induced by TGF-beta and is critical for TGF-beta dependent cell cycle arrest.	[1,2,3,4,5,6]
**Dlk1 (Pref1)**	Paternal	Encodes a delta-like ligand that can be either soluble or membrane bound. It is thought to inhibit Notch signaling and plays a role in regulating differentiation of adipocytes and mesenchymal stem cells.	KO: Growth restriction, accelerated adiposity<br>OE: "Callipyge" phenotype	Expressed in human $CD34^{+}$ hematopoietic stem cells and upregulated in some cases of hematopoietic malignancy, particularly myelodysplasia and myeloid leukemia. Expressed by stromal cells that support hematopoiesis, suggesting that it plays a role in the interactions between stem cells and their niche.	[7,8,9,10,11,12,13]
**Grb10**	Maternal	Adapter protein for receptor tyrosine kinases, with roles in insulin and insulin-like growth factor signaling. Conflicting reports regarding repression or enhancement of receptor signaling, likely dependent on cellular context.	KO: Overgrowth, increased muscle mass / reduced adiposity, altered glucose homeostasis	Binds to the chimeric BCR-ABL tyrosine kinase expressed in chronic myelogenous leukemia. May be involved signaling from the c-Kit tyrosine kinase receptor.	[14,15,16,17, 18]
**Gtl2 (Meg3)**	Maternal	Non-coding RNA that harbors two miRNA clusters and a snoRNA cluster. Part of a large reciprocally imprinted domain including *Dlk1*, *anti-Rtl1*, and *Dio3*. Several miRNAs from this cluster are downregulated in ovarian cancer, suggesting a possible tumor suppressor role.	KO: Depends on parent of origin	?	[19,20,21,22, 23,24]
**H19**	Maternal	Non-coding RNA that harbors miR-675. The precise targets are unclear. Likely functions as a tumor suppressor, but conflicting reports. H19 transcript is upregulated during cell proliferation (possibly due to reduced miRNA processing).	KO: Confounded by effects on Igf2 expression;<br>OE: Repressed growth	Downregulated in CD34+/CD33- bone marrow cells from patients with Polycythemia vera and downregulated in several different leukemias and myeloproliferative disorders.	[25,26,27,28, 29,30,31,32,33]
**Igf2**	Paternal	Secreted growth factor that promotes fetal growth. Likely involved in both paracrine/endocrine signaling. Upregulated in many different cancers, in many cases due to loss of imprinting.	KO: Growth restriction, complicated by effects on placental function<br>OE: Overgrowth, multiple malformations	Growth factor for fetal hematopoietic stem cells. Downregulated in several different leukemias.	[33,34,35,36, 37,38,39]
**Mest (Peg1)**	Paternal	Expressed in embryonic mesoderm, mesodermal derivatives, and the central nervous system. May have enzymatic function as an epoxide hydrolase based on protein homology. Upregulated in obese adipose tissue.	KO: Intrauterine growth restriction	?	[40,41,42]
**Ndn**	Paternal	MAGE protein, initially discovered in post-mitotic neurons thought to arrest growth and regulate apoptosis. Multiple biochemical interactions. Implicated in differentiation of neuronal and mesenchymal tissues.	KO: Conflicting data, early post-natal lethality due to respiratory failure, possible strain differences	Likely transcriptional target of p53 and plays modest role in regulating HSC quiescence, primarily during regeneration.	[43,44,45,46, 47,48,49,50,51,52]
**Peg3 (Pw1)**	Paternal	Zinc-finger transcription factor expressed in the embryonic mesoderm and brain, plays a role in TNF-alpha/NF-kB signaling and p53-mediated apoptosis. Implicated in muscle stem cell function.	KO: Intrauterine growth restriction	?	[53,54,55,56, 57]
**Plagl1 (Zac1 / Lot1)**	Paternal	Zinc-finger transcription factor that inhibits the growth of tumor cells in vitro through repression of cell cycle and induction of apoptosis. Frequently deleted or down-regulated in cancer, co-regulates p53-responsive promoters.	KO: Intrauterine growth restriction	?	[58,59,60,61, 62,63]

References associated with Table S1

1. Pateras IS, Apostolopoulou K, Niforou K, Kotsinas A, Gorgoulis VG (2009) p57KIP2: "Kip"ing the cell under control. Molecular cancer research : MCR 7: 1902-1919.
2. Vlachos P, Joseph B (2009) The Cdk inhibitor p57(Kip2) controls LIM-kinase 1 activity and regulates actin cytoskeleton dynamics. Oncogene 28: 4175-4188.
3. Zhang P, Liegeois NJ, Wong C, Finegold M, Hou H, et al. (1997) Altered cell differentiation and proliferation in mice lacking p57KIP2 indicates a role in Beckwith-Wiedemann syndrome. Nature 387: 151-158.
4. Passegue E, Wagers AJ, Giuriato S, Anderson WC, Weissman IL (2005) Global analysis of proliferation and cell cycle gene expression in the regulation of hematopoietic stem and progenitor cell fates. The Journal of experimental medicine 202: 1599-1611.
5. Umemoto T, Yamato M, Nishida K, Yang J, Tano Y, et al. (2005) p57Kip2 is expressed in quiescent mouse bone marrow side population cells. Biochemical and biophysical research communications 337: 14-21.
6. Scandura JM, Boccuni P, Massague J, Nimer SD (2004) Transforming growth factor beta-induced cell cycle arrest of human hematopoietic cells requires p57KIP2 up-regulation. Proc Natl Acad Sci U S A 101: 15231-15236.
7. Baladron V, Ruiz-Hidalgo MJ, Nueda ML, Diaz-Guerra MJ, Garcia-Ramirez JJ, et al. (2005) dlk acts as a negative regulator of Notch1 activation through interactions with specific EGF-like repeats. Experimental cell research 303: 343-359.
8. Abdallah BM, Boissy P, Tan Q, Dahlgaard J, Traustadottir GA, et al. (2007) dlk1/FA1 regulates the function of human bone marrow mesenchymal stem cells by modulating gene expression of pro-inflammatory cytokines and immune response-related factors. The Journal of biological chemistry 282: 7339-7351.
9. Davis E, Jensen CH, Schroder HD, Farnir F, Shay-Hadfield T, et al. (2004) Ectopic expression of DLK1 protein in skeletal muscle of padumnal heterozygotes causes the callipyge phenotype. Current biology : CB 14: 1858-1862.
10. Nueda ML, Baladron V, Sanchez-Solana B, Ballesteros MA, Laborda J (2007) The EGF-like protein dlk1 inhibits notch signaling and potentiates adipogenesis of mesenchymal cells. Journal of molecular biology 367: 1281-1293.
11. Moon YS, Smas CM, Lee K, Villena JA, Kim KH, et al. (2002) Mice lacking paternally expressed Pref-1/Dlk1 display growth retardation and accelerated adiposity. Molecular and cellular biology 22: 5585-5592.
12. Sakajiri S, O'Kelly J, Yin D, Miller CW, Hofmann WK, et al. (2005) Dlk1 in normal and abnormal hematopoiesis. Leukemia 19: 1404-1410.
13. Qi X, Chen Z, Liu D, Cen J, Gu M (2008) Expression of Dlk1 gene in myelodysplastic syndrome determined by microarray, and its effects on leukemia cells. International journal of molecular medicine 22: 61-68.
14. Riedel H (2004) Grb10 exceeding the boundaries of a common signaling adapter. Frontiers in bioscience : a journal and virtual library 9: 603-618.
15. Charalambous M, Smith FM, Bennett WR, Crew TE, Mackenzie F, et al. (2003) Disruption of the imprinted Grb10 gene leads to disproportionate overgrowth by an Igf2-independent mechanism. Proceedings of the National Academy of Sciences of the United States of America 100: 8292-8297.

16. Smith FM, Holt LJ, Garfield AS, Charalambous M, Koumanov F, et al. (2007) Mice with a disruption of the imprinted Grb10 gene exhibit altered body composition, glucose homeostasis, and insulin signaling during postnatal life. Molecular and cellular biology 27: 5871-5886.
17. Bai RY, Jahn T, Schrem S, Munzert G, Weidner KM, et al. (1998) The SH2-containing adapter protein GRB10 interacts with BCR-ABL. Oncogene 17: 941-948.
18. Jahn T, Seipel P, Urschel S, Peschel C, Duyster J (2002) Role for the adaptor protein Grb10 in the activation of Akt. Molecular and cellular biology 22: 979-991.
19. Cavaille J, Seitz H, Paulsen M, Ferguson-Smith AC, Bachellerie JP (2002) Identification of tandemly-repeated C/D snoRNA genes at the imprinted human 14q32 domain reminiscent of those at the Prader-Willi/Angelman syndrome region. Human molecular genetics 11: 1527-1538.
20. Seitz H, Royo H, Bortolin ML, Lin SP, Ferguson-Smith AC, et al. (2004) A large imprinted microRNA gene cluster at the mouse Dlk1-Gtl2 domain. Genome Res 14: 1741-1748.
21. Kircher M, Bock C, Paulsen M (2008) Structural conservation versus functional divergence of maternally expressed microRNAs in the Dlk1/Gtl2 imprinting region. BMC genomics 9: 346.
22. Zhang L, Volinia S, Bonome T, Calin GA, Greshock J, et al. (2008) Genomic and epigenetic alterations deregulate microRNA expression in human epithelial ovarian cancer. Proceedings of the National Academy of Sciences of the United States of America 105: 7004-7009.
23. Steshina EY, Carr MS, Glick EA, Yevtodiyenko A, Appelbe OK, et al. (2006) Loss of imprinting at the Dlk1-Gtl2 locus caused by insertional mutagenesis in the Gtl2 5' region. BMC genetics 7: 44.
24. Takahashi N, Okamoto A, Kobayashi R, Shirai M, Obata Y, et al. (2009) Deletion of Gtl2, imprinted non-coding RNA, with its differentially methylated region induces lethal parent-origin-dependent defects in mice. Human molecular genetics 18: 1879-1888.
25. Hao Y, Crenshaw T, Moulton T, Newcomb E, Tycko B (1993) Tumour-suppressor activity of H19 RNA. Nature 365: 764-767.
26. Ariel I, Ayesh S, Perlman EJ, Pizov G, Tanos V, et al. (1997) The product of the imprinted H19 gene is an oncofetal RNA. Molecular pathology : MP 50: 34-44.
27. Cai X, Cullen BR (2007) The imprinted H19 noncoding RNA is a primary microRNA precursor. Rna 13: 313-316.
28. Looijenga LH, Verkerk AJ, De Groot N, Hochberg AA, Oosterhuis JW (1997) H19 in normal development and neoplasia. Molecular reproduction and development 46: 419-439.
29. Yamamoto Y, Nishikawa Y, Tokairin T, Omori Y, Enomoto K (2004) Increased expression of H19 non-coding mRNA follows hepatocyte proliferation in the rat and mouse. Journal of hepatology 40: 808-814.
30. Milligan L, Antoine E, Bisbal C, Weber M, Brunel C, et al. (2000) H19 gene expression is up-regulated exclusively by stabilization of the RNA during muscle cell differentiation. Oncogene 19: 5810-5816.
31. Nunez C, Bashein AM, Brunet CL, Hoyland JA, Freemont AJ, et al. (2000) Expression of the imprinted tumour-suppressor gene H19 is tightly regulated during normal haematopoiesis and is reduced in haematopoietic precursors of patients with the myeloproliferative disease polycythaemia vera. J Pathol 190: 61-68.

32. Bock O, Schlue J, Kreipe H (2003) Reduced expression of H19 in bone marrow cells from chronic myeloproliferative disorders. Leukemia : official journal of the Leukemia Society of America, Leukemia Research Fund, UK 17: 815-816.
33. Tessema M, Langer F, Bock O, Seltsam A, Metzig K, et al. (2005) Down-regulation of the IGF-2/H19 locus during normal and malignant hematopoiesis is independent of the imprinting pattern. Int J Oncol 26: 499-507.
34. Chao W, D'Amore PA (2008) IGF2: epigenetic regulation and role in development and disease. Cytokine & growth factor reviews 19: 111-120.
35. Rodriguez S, Gaunt TR, Day IN (2007) Molecular genetics of human growth hormone, insulin-like growth factors and their pathways in common disease. Human genetics 122: 1-21.
36. Kaneda A, Feinberg AP (2005) Loss of imprinting of IGF2: a common epigenetic modifier of intestinal tumor risk. Cancer research 65: 11236-11240.
37. Constancia M, Hemberger M, Hughes J, Dean W, Ferguson-Smith A, et al. (2002) Placental-specific IGF-II is a major modulator of placental and fetal growth. Nature 417: 945-948.
38. Sun FL, Dean WL, Kelsey G, Allen ND, Reik W (1997) Transactivation of Igf2 in a mouse model of Beckwith-Wiedemann syndrome. Nature 389: 809-815.
39. Zhang CC, Lodish HF (2004) Insulin-like growth factor 2 expressed in a novel fetal liver cell population is a growth factor for hematopoietic stem cells. Blood 103: 2513-2521.
40. Kaneko-Ishino T, Kuroiwa Y, Miyoshi N, Kohda T, Suzuki R, et al. (1995) Peg1/Mest imprinted gene on chromosome 6 identified by cDNA subtraction hybridization. Nat Genet 11: 52-59.
41. Kamei Y, Suganami T, Kohda T, Ishino F, Yasuda K, et al. (2007) Peg1/Mest in obese adipose tissue is expressed from the paternal allele in an isoform-specific manner. FEBS letters 581: 91-96.
42. Lefebvre L, Viville S, Barton SC, Ishino F, Keverne EB, et al. (1998) Abnormal maternal behaviour and growth retardation associated with loss of the imprinted gene Mest. Nature genetics 20: 163-169.
43. Hayashi Y, Matsuyama K, Takagi K, Sugiura H, Yoshikawa K (1995) Arrest of cell growth by necdin, a nuclear protein expressed in postmitotic neurons. Biochemical and biophysical research communications 213: 317-324.
44. Barker PA, Salehi A (2002) The MAGE proteins: emerging roles in cell cycle progression, apoptosis, and neurogenetic disease. Journal of neuroscience research 67: 705-712.
45. Brunelli S, Tagliafico E, De Angelis FG, Tonlorenzi R, Baesso S, et al. (2004) Msx2 and necdin combined activities are required for smooth muscle differentiation in mesoangioblast stem cells. Circulation research 94: 1571-1578.
46. Tseng YH, Butte AJ, Kokkotou E, Yechoor VK, Taniguchi CM, et al. (2005) Prediction of preadipocyte differentiation by gene expression reveals role of insulin receptor substrates and necdin. Nature cell biology 7: 601-611.
47. Deponti D, Francois S, Baesso S, Sciorati C, Innocenzi A, et al. (2007) Necdin mediates skeletal muscle regeneration by promoting myoblast survival and differentiation. The Journal of cell biology 179: 305-319.
48. Kurita M, Kuwajima T, Nishimura I, Yoshikawa K (2006) Necdin downregulates CDC2 expression to attenuate neuronal apoptosis. The Journal of neuroscience : the official journal of the Society for Neuroscience 26: 12003-12013.

49. Tsai TF, Armstrong D, Beaudet AL (1999) Necdin-deficient mice do not show lethality or the obesity and infertility of Prader-Willi syndrome. Nature genetics 22: 15-16.
50. Gerard M, Hernandez L, Wevrick R, Stewart CL (1999) Disruption of the mouse necdin gene results in early post-natal lethality. Nature genetics 23: 199-202.
51. Liu Y, Elf SE, Miyata Y, Sashida G, Huang G, et al. (2009) p53 regulates hematopoietic stem cell quiescence. Cell Stem Cell 4: 37-48.
52. Kubota Y, Osawa M, Jakt LM, Yoshikawa K, Nishikawa S (2009) Necdin restricts proliferation of hematopoietic stem cells during hematopoietic regeneration. Blood 114: 4383-4392.
53. Relaix F, Weng X, Marazzi G, Yang E, Copeland N, et al. (1996) Pw1, a novel zinc finger gene implicated in the myogenic and neuronal lineages. Developmental biology 177: 383-396.
54. Relaix F, Wei XJ, Wu X, Sassoon DA (1998) Peg3/Pw1 is an imprinted gene involved in the TNF-NFkappaB signal transduction pathway. Nature genetics 18: 287-291.
55. Relaix F, Wei X, Li W, Pan J, Lin Y, et al. (2000) Pw1/Peg3 is a potential cell death mediator and cooperates with Siah1a in p53-mediated apoptosis. Proc Natl Acad Sci U S A 97: 2105-2110.
56. Deng Y, Wu X (2000) Peg3/Pw1 promotes p53-mediated apoptosis by inducing Bax translocation from cytosol to mitochondria. Proceedings of the National Academy of Sciences of the United States of America 97: 12050-12055.
57. Nicolas N, Marazzi G, Kelley K, Sassoon D (2005) Embryonic deregulation of muscle stress signaling pathways leads to altered postnatal stem cell behavior and a failure in postnatal muscle growth. Developmental biology 281: 171-183.
58. Spengler D, Villalba M, Hoffmann A, Pantaloni C, Houssami S, et al. (1997) Regulation of apoptosis and cell cycle arrest by Zac1, a novel zinc finger protein expressed in the pituitary gland and the brain. The EMBO journal 16: 2814-2825.
59. Varrault A, Ciani E, Apiou F, Bilanges B, Hoffmann A, et al. (1998) hZAC encodes a zinc finger protein with antiproliferative properties and maps to a chromosomal region frequently lost in cancer. Proceedings of the National Academy of Sciences of the United States of America 95: 8835-8840.
60. Varrault A, Gueydan C, Delalbre A, Bellmann A, Houssami S, et al. (2006) Zac1 regulates an imprinted gene network critically involved in the control of embryonic growth. Dev Cell 11: 711-722.
61. Abdollahi A (2007) LOT1 (ZAC1/PLAGL1) and its family members: mechanisms and functions. Journal of cellular physiology 210: 16-25.
62. Basyuk E, Coulon V, Le Digarcher A, Coisy-Quivy M, Moles JP, et al. (2005) The candidate tumor suppressor gene ZAC is involved in keratinocyte differentiation and its expression is lost in basal cell carcinomas. Molecular cancer research : MCR 3: 483-492.
63. Huang SM, Schonthal AH, Stallcup MR (2001) Enhancement of p53-dependent gene activation by the transcriptional coactivator Zac1. Oncogene 20: 2134-2143.
