# Supplementary material for: Imprinted Genes That Regulate Early Mammalian Growth Are Coexpressed in Somatic Stem Cells
Source: PLoS One. 2011 Oct 19;6(10):e26410. doi: 10.1371/journal.pone.0026410 (PMC3198398; doi:10.1371/journal.pone.0026410)
Supplement: Table S2 — SNPs used for analysis of monoallelic expression in F1 progeny of Castaneous male and C57BL/6 female mice. (PDF) [file pone.0026410.s004.pdf]

Table S2: SNPs used for analysis of monoallelic expression in F1 progeny of Castaneous male and C57BL/6 female mice

Gene	SNP	Position	BL/6 allele	Cast allele	Primers	Expressed allele
Dlk1	NES17644873	chr12:110,698,537	T	C	(F) AGTATAACAGCGGCGAGGAG<br>(R) AGCTCTAAGGAACCCCGGTA	C
Gtl2	NES17649632	chr12:110,797,703	T	C	(F) CACAGAGCACACAGTCACCA<br>(R) GGATTAACGATCCACCAGCA	T
	NES17649638	chr12:110,797,931	A	C		A
H19	Szabo & Mann[68]	chr7:149,761,689	A	C	(F) AGGAATCTGCTCCAAGGTGA<br>(R) GCATTGCCAAAGAGGTTTACA	A
Igf2	Szabo & Mann[68]	chr7:149,839,519	C	T	(F) TCCGAGAGGGACGTGTCTAC<br>(R) GGGTGTCAATTGGGTTGTTT	T
Peg3	NES16164957	chr7:6,659,876	G	A	(F) CAAAGACTTTCACTCTCATCACA<br>(R) GGCAGGAATCATTATGTATAGGG	A
	NES16164959	chr7:6,659,966	T	G		G

SNPs are named according to Perlegen IDs (http://mouse.perlegen.com/mouse/browser.html) as previously reported [1]. Positions are based on the mm9 (NCBI37) assembly of the mouse genome. Note that the alleles listed here reflect the (+) strand, whereas the sequencing traces for H19, Igf2 and Peg3 (shown in Fig. S1) reflect the (-) strand.

Reference for table S2

1. Frazer KA, Eskin E, Kang HM, Bogue MA, Hinds DA, et al. (2007) A sequence-based variation map of 8.27 million SNPs in inbred mouse strains. Nature 448: 1050-1053.
